# Statistically Optimum HKUST-1 Synthesized by Room Temperature Coordination Modulation Method for the Adsorption of Crystal Violet Dye

**DOI:** 10.3390/molecules26216430

**Published:** 2021-10-25

**Authors:** Christian J. Wijaya, Suryadi Ismadji, Hakun W. Aparamarta, Setiyo Gunawan

**Affiliations:** 1 Department of Chemical Engineering, Faculty of Industrial Technology and Systems Engineering, Institut Teknologi Sepuluh Nopember, Keputih Sukolilo, Surabaya 60111, Indonesia; ch.julius7@gmail.com (C.J.W.); hakun2397@gmail.com (H.W.A.); 2 Department of Chemical Engineering, Widya Mandala Surabaya Catholic University, Kalijudan 37, Surabaya 60114, Indonesia; suryadiismadji@yahoo.com; 3 Department of Chemical Engineering, National Taiwan University of Science and Technology, 43 Keelung Road, Sec. 4, Taipei 10607, Taiwan

**Keywords:** coordination modulation synthesis, dye adsorption, healthy, HKUST-1, metal-organic frameworks, optimization

## Abstract

Due to its excellency and versatility, many synthesis methods and conditions were developed to produce HKUST-1 ([Cu_3_(BTC)_2_(H_2_O)_3_]_n_). However, the diversity of HKUST-1 was actually generated both in terms of characteristics and morphologies. Hence, the consistency of HKUST-1 characteristics and morphologies needs to be maintained. The statistical analysis and optimization provide features to determine the best synthesis condition. Here, a room-temperature coordination modulation method was proposed to maintain the morphology of HKUST-1 while reducing energy consumption. In addition, response surface methodology (RSM) was used to demonstrate the statistical analysis and optimization of the synthesis of HKUST-1. The molar ratio of ligand to metal, reaction time, and acetic acid concentration were studied to determine their effects on HKUST-1. The optimum HKUST-1 was obtained by the synthesis with a molar ratio of ligand to metal of 0.4703 for 27.2 h using 5% *v*/*v* acetic acid concentration. The statistical analysis performed a good agreement with the experimental data and showed the significance of three desired parameters on HKUST-1. The optimum HKUST-1 had the adsorption capacity of 1005.22 mg/g with a removal efficiency of 92.31% towards CV dye. It could be reused up to 5 cycles with insignificant decrease in performance.

## 1. Introduction

Porous materials are widely needed in various applications, such as adsorbents, catalysts, energy storages, and medical fields. Therefore, many researchers have developed various porous materials with certain characteristics and specifications according to their application. In the last few decades, metal-organic frameworks (MOFs) have become one of the most widely developed and applied porous materials. MOFs are composed of metal clusters and organic ligands through coordination bonds to perform various frameworks. In the framework, metal clusters play a role as inorganic parts and act as “lateral”, while organic ligands act as “joint” [1]. The utilization of MOFs in various applications is due to their large surface area, high and tunable porosity, ease of modification, and abundant active sites [2,3]. It is very potential for selective adsorption and degradation systems [4,5,6,7]. MOFs have a flexible synthesis process, where different types of metals, ligands, synthesis methods, and operating conditions can generate various morphologies and characteristics of MOFs [8,9]. Hence, there are 99,075 structures of MOFs reported and recorded in the Cambridge Structural Database (CSD) as of August 2020 and will continue to grow along with the high needs in various applications [10].

Copper (Cu)-based MOFs are one type of MOF that continue to be widely developed due to their outstanding physical and chemical characteristics. In the family of Cu-based MOFs, Hong Kong University of Science and Technology-1 (HKUST-1, [Cu_3_(BTC)_2_(H_2_O)_3_]_n_), which is also known as MOF-199, is one of the excellent MOFs constructed by Cu clusters and benzene-1,3,5-tricarboxylic acid (H_3_BTC) in a paddle-wheel secondary building unit [11,12]. HKUST-1 has a large surface area, high porosity, high crystallinity, good chemical and thermal stability, easy functionalization, and abundant unsaturated metal sites [3,8,9,13,14]. Due to its excellent characteristics, HKUST-1 can be used in many applications, either as a precursor [15], adsorbent [16,17,18], carrier [19], storage [20], catalyst [21], or membrane [22]. The synthesis of HKUST-1 has been carried out through many methods, such as hydro/solvothermal [12,23], atmospheric-pressure synthesis [8,24], mechanochemical [25,26,27], sonochemical [28,29,30], electrochemical [31,32,33], microwave-assisted synthesis [34,35], sol-gel synthesis [36], dry gel conversion [37], and solvent-free vacuum growth [38]. Here, all synthesis methods have their advantages, but they also have drawbacks at different points, such as long synthesis times, high energy consumptions, or even poorer product characteristics. So far, the hydro/solvothermal method is the most preferred method to synthesize MOFs, including HKUST-1. This method can produce HKUST-1 with better quality in terms of physical and chemical characteristics. However, the hydro/solvothermal method requires a longer reaction time compared to other methods. Moreover, a lot of energy is consumed because of the combination of high temperature, high pressure, and long reaction time used in the hydro/solvothermal method. In the synthesis of MOFs, high energy consumption is not only an issue in the hydro/solvothermal method but almost in all other methods. Hence, room-temperature synthesis is developed as a solution to overcome this issue.

Room-temperature synthesis has been used to generate HKUST-1 in several previous studies [13,14,39,40,41,42,43]. This synthesis method continues to be developed because it is easier and more energy efficient but it still produces HKUST-1 with similar HKUST-1 characteristics to other synthesis methods. However, each research using the room-temperature synthesis method was carried out under various conditions in terms of precursor ratios, solvent types, and reaction times. In terms of solvent type, water, ethanol, dimethylformamide (DMF), ionic liquids, or solvent combination could be used previously in the room-temperature synthesis method [14,39,42,43]. Besides that, this method was done at various reaction times in previous researches, such as in 10 min [41], 30 min [14], 2 h [40], 12 h [13], or even 24 h [43]. This diversity has an impact on the HKUST-1 product, where it will differ in physical and chemical characteristics. As evidence, HKUST-1 has various morphologies due to different synthesis conditions, such as octahedrons [14], cubes [13], cuboctahedrons [40], hexagonal polyhedrons [23], rod-like shapes [21], irregular-layered sheets [43], agglomerates [11], or even monoliths [12]. This uncertain morphology can be overcome by the coordination modulation method using additional modulators or chemicals to maintain crystal growth of HKUST-1, such as acetic acid [44,45], nitric acid [46,47], sodium bicarbonate [13,48], sodium acetate, sodium formate, and triethylamine [49]. Further than this, a statistical optimization of the HKUST-1 synthesis process needs to be studied to overcome the diversity of synthesis conditions so that the optimum HKUST-1 can be generated and further utilized. To the best of our knowledge, this kind of statistical optimization has never been done in previous studies.

In this study, HKUST-1 was synthesized using the room-temperature coordination modulation method with the addition of acetic acid as a modulator. A statistical optimization was carried out using the central composite design (CCD) of response surface methodology (RSM) in Minitab software to determine the optimum synthesis condition. Here, three parameters were varied to investigate their effects on HKUST-1, namely the molar ratio of ligand to metal, reaction time, and acetic acid concentration. Next, HKUST-1 synthesized using various combinations of parameters was used to remove crystal violet (CV) dye in an aqueous solution. Here, CV dye was chosen because of the high adsorption capacity of HKUST-1 towards CV dye compared to other dyes from the preliminary studies. In the statistical study, the amount of CV dye adsorbed onto HKUST-1 (q) was used as the measurable statistical response. Furthermore, the CV dye removal using the optimum HKUST-1 was studied in terms of adsorption capacity, removal efficiency, and reusability.

## 2. Results

### 2.1. Synthesis of HKUST-1

#### 2.1.1. Statistical Studies

**Figure 1:**
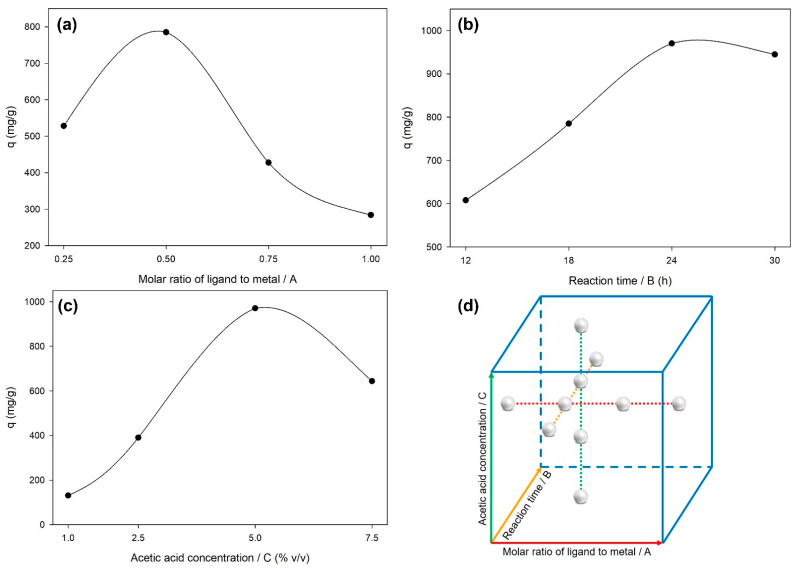
Simulation of levels of (**a**) molar ratio of ligand to metal, (**b**) reaction time, and (**c**) acetic acid concentration towards the response by (**d**) OFAT method.

The synthesis of HKUST-1 was investigated by CCD of RSM using three independent parameters, such as the molar ratio of ligand to metal (A), reaction time (B), and acetic acid concentration (C). Initially, the levels of these parameters were determined based on the one-factor-at-a-time (OFAT) experiments. In the OFAT experiments, two of three parameters were held at a certain level, while another level was varied as designed in Figure 1d. Figure 1a shows the effect of the molar ratio of ligand to metal on q, where the peak of q is obtained between 0.25 to 0.75. It indicates that the molar ratio of ligand to metal affects the characteristics of HKUST-1. At a certain value of the molar ratio of ligand to metal, the synthesized HKUST-1 will have a higher adsorption capacity of CV dye. After the molar ratio of ligand to metal of 0.5, q decreases because the residue of excess ligand used in the synthesis of HKUST-1 was adsorbed on HKUST-1 and blocked its surface-active sites. Figure 1b exhibits the effect of reaction time on q, where the peak of q is obtained between 18 to 30 h. Here, q increases along with the reaction time until the reaction time is about 27 h and then q gradually decreases. This can be interpreted that the reaction time affects the crystal growth of HKUST-1. The highest q can be obtained with HKUST-1, which has an immature crystal growth. However, excessive crystal growth can cause agglomerations and morphological changes of HKUST-1, which is indicated by a gradual decrease in q. Figure 1c presents the effect of acetic acid concentration on q, where the peak of q is obtained between 2.5 to 7.5% *v*/*v*. Here, the addition of acetic acid as a modulator with a certain concentration controls the growth of the HKUST-1 crystal nuclei into HKUST-1 crystals with a well-defined structure and morphology. From these OFAT experiments, the level range of each parameter was obtained and then used in further statistical analysis with RSM to study the effects of the interactions of the parameters and obtain the optimum condition for the synthesis of HKUST-1.

Furthermore, the statistical analysis using RSM was performed for the synthesis of HKUST-1 under three independent parameters and five levels for each parameter consisting of three defined levels obtained from OFAT experiments and two extreme levels. Sixty runs including three replications were carried out according to the CCD of RSM designed in Minitab software. Table 1 shows the values of actual and predicted q for each combined parameter, where both have closed values and similar trends. This means that CCD is a suitable model for statistical studies of the synthesis of HKUST-1 with the specified parameters. Here, the predicted q was calculated from the polynomial Equation (1) obtained and expressed below:

(1) $$q_{predicted}=972.02-45.05A+83.71B+85.27C-193.36A^{2}-81.47B^{2}-246.53C^{2}-4.38AC+15.27BC$$

where $q_{predicted}$ is the predicted value of q from statistical analysis, while A, B, and C are the coded values of three independent parameters. This equation excludes the term of the interaction between the molar ratio of ligand to metal and reaction time because it is not significant (*p*-value > 0.05) based on an ANOVA evaluation as shown in Table 2.

Table 2 shows the result of ANOVA included within the statistical analysis using CCD of RSM. ANOVA result provides an interpretation of significance between the model, parameters, interactions, and statistical error. The significance is determined based on a *p*-value below 0.05. As shown in Table 2, the two-way interaction between the molar ratio of ligand to metal and reaction time has a *p*-value higher than 0.05, which is not significant to the response. However, the other linear, quadratic, and two-way interaction parameters indicate a significant effect on the response. The fitness of the CCD of RSM can be proven by the insignificance of the lack-of-fit. This indicates that the error does not have a significant effect on the statistical analysis and the CCD of RSM can be used to model and predict the statistical response. Other than that, the high values of $R^{2}$ (99.92%), adjusted $R^{2}$ (99.90%), and predicted $R^{2}$ (99.87%) show consistent results where the statistical model used is well fitted to the experimental data.

**Figure 2:**
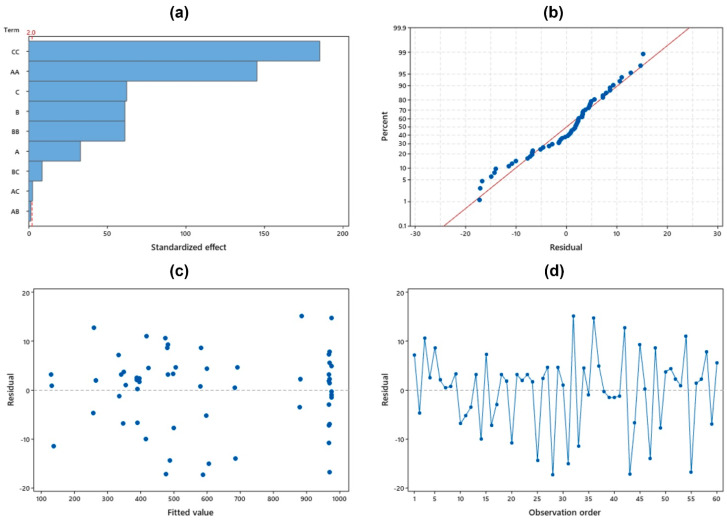
Condition of statistical study interpreted using (**a**) Pareto chart, (**b**) normal probability plot, (**c**) residuals versus fits plot, and (**d**) residuals versus order plot.

Furthermore, the significance of each parameter is depicted in a Pareto chart as shown in Figure 2a and is distinguished by a red dashed line. As presented in Figure 2b, the normal probability plot shows that the residual points are located around the red straight diagonal line, where it can be interpreted that the residuals are normally distributed. The residuals versus fits plot shows a random and unrecognizable pattern in either the positive or negative residual range as figured in Figure 2c. This constant of variance indicated by this random pattern convinces that the model used is valid. The independence of residuals is shown through the unseen trend in the residuals versus order plot as plotted in Figure 2d. The above interpretations confirm that the CCD of RSM is well fitted to the experimental data and can be used to explore the effects of these three independent parameters in the synthesis of HKUST-1.

**Figure 3:**
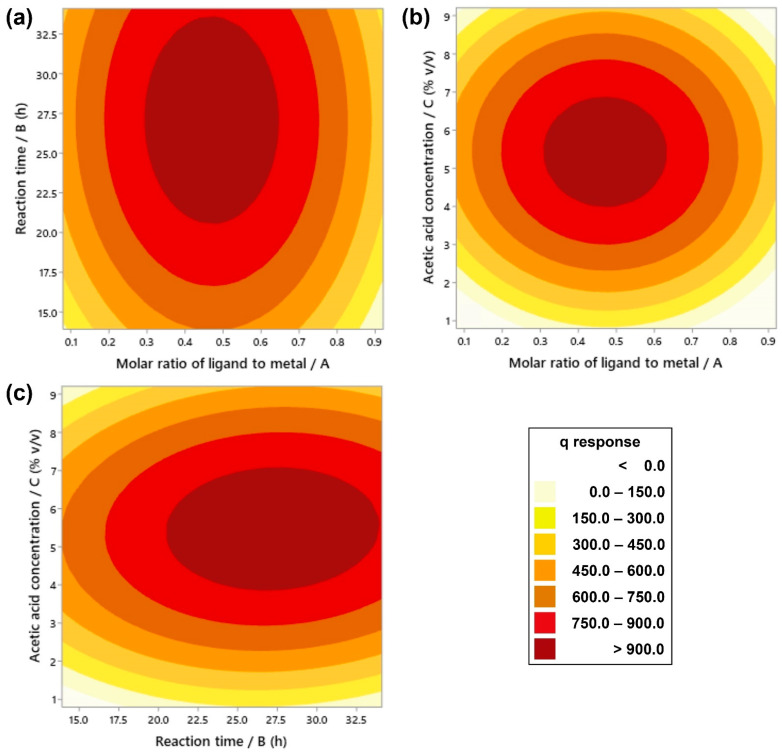
Contour plots showing the effects of two independent parameters by holding another parameter at the middle level: the effects of (**a**) molar ratio of ligand to metal and reaction time, (**b**) molar ratio of ligand to metal and acetic acid concentration, and (**c**) reaction time and acetic acid concentration.

Figure 3 shows the 2D contour plots that evaluate the interaction effects of two parameters with respect to the statistical response while another parameter is held at the middle level. The increase of q value is expressed in terms of the area from a lighter to a darker color. As plotted in Figure 3, the q value can reach over 900 mg/g as represented in the darkest red area. Actually, the highest *q* value can be detected at the middle point of the darkest red area, where the molar ratio of ligand to metal, reaction time, and acetic acid concentration are around 0.45, 27.5 h, and 5.5% *v*/*v*, respectively. However, more precise parameter values were further analyzed using the optimization function of RSM in Minitab software.

#### 2.1.2. Optimum Condition Localization and Its Validation 

**Figure 4:**
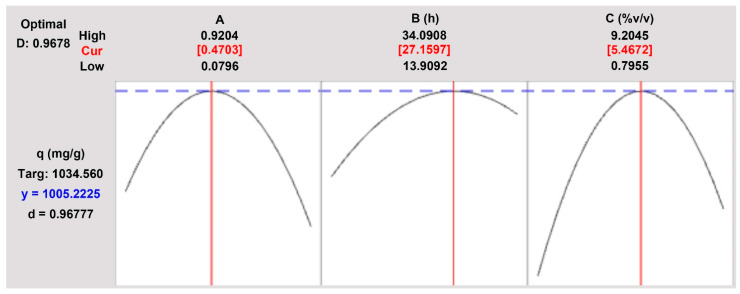
Optimization plot.

In RSM, optimization has an important role in generating the best condition that gives the highest response, wherein this case it is used to find out the optimum level of each independent parameter in the synthesis of HKUST-1. In the previous studies, HKUST-1 has been tried to adsorb some dyes in aqueous solution with a fairly high removal efficiency, such as congo red (>90%) [50], malachite green (83.4%), eosin yellow (94.9%) [51], and methylene blue (~95%) [52]. Hence, this study targeted the optimum HKUST-1 which could adsorb crystal violet dye in an aqueous solution with a removal efficiency close to or higher than previous studies. As shown in Figure 4, the optimum HKUST-1 can be obtained by synthesis with a molar ratio of ligand to metal of 0.4703 for 27.2 h using 5% *v*/*v* acetic acid concentration. Based on the optimization study, the q value as a response was predicted to reach 1005.22 mg/g on CV dye adsorption using the optimum HKUST-1, where the removal efficiency was equal to 92.31%. This result also exceeded the adsorption capacity of other MOFs towards CV, such as ZIF-L with 823.02 mg/g [53], Zn-FODC with 54.50 mg/g [54], and H_2_*dtoa*Cu with 165.83 mg/g [55]. This optimization gave a satisfactory result as proven by the high composite desirability of 0.9678. However, it still needed to be validated for ensuring the accuracy and precision of the optimization result.

Further, the synthesis of HKUST-1 was re-performed three times using the optimum level of parameters obtained from the optimization study. Then, it also was used to adsorb CV dye in an aqueous solution with the same initial concentration. As presented in Table 3, the actual optimum q values are not significantly different to the predicted optimum q value. As a result, the mean optimum *q* value reached 977.99 ± 6.51 mg/g with a mean error of 2.71 ± 0.65% against the predicted optimum *q* value. Hence, this low deviation standard of q value indicates the precision of these experiments, while the optimization study is valid due to the low error percentage obtained from these validation runs.

### 2.2. Characterization of HKUST-1

**Figure 5:**
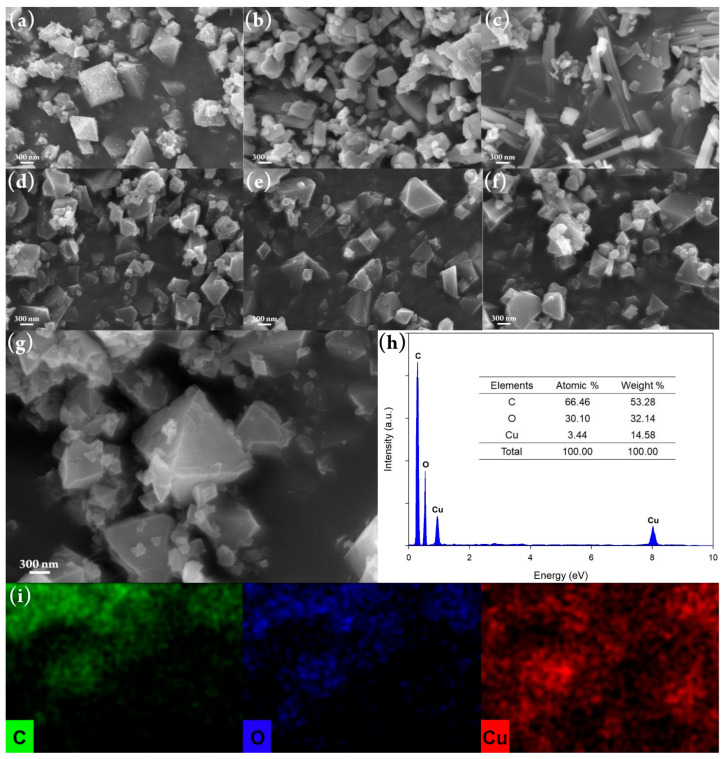
SEM images of HKUST-1 synthesized with various molar ratios of ligand to metal: (**a**) 0.25, (**b**) 0.75, and (**c**) 1.00; synthesized with various acetic acid concentrations: (**d**) 2.5% *v*/*v*, (**e**) 7.5% *v*/*v*, and (**f**) 10.0% *v*/*v*; (**g**) synthesized under optimum condition; (**h**) EDX spectra of HKUST-1; and (**i**) EDX elemental mapping of HKUST-1.

Several characterizations, such as SEM, EDX, XRD, FTIR, and TGA were performed to investigate the HKUST-1 characteristics. As depicted in Figure 5, the morphology of HKUST-1 is dependent on the independent parameters used in the synthesis process. Here, the higher molar ratio of ligand to metal used leads to a morphological transformation from octahedrons to rod-like shapes, as shown in Figure 5a–c. On the contrary, Figure 5d–f shows the presence of octahedral HKUST-1 due to the addition of acetic acid as a modulator.

**Figure 6:**
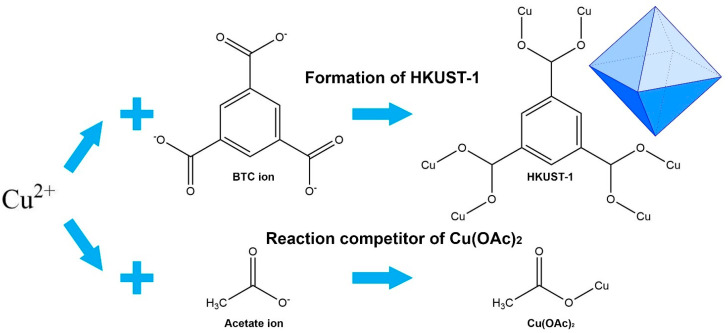
Coordination modulation mechanism of HKUST-1 synthesis.

Indeed, the concentration of acetic acid here greatly affects the morphological uniformity, but this still proves that the modulator can help to maintain the octahedron morphology. Here, the presence of modulators, such as carboxylic acids, carboxylate salts, other acids, and bases, controls the coordination equilibrium during the crystal formation of HKUST-1 [56]. Moreover, it can also control the crystal size of HKUST-1, where the nucleation rate is very sensitive to pH conditions which can be regulated through the addition of a modulator [49]. Here, the addition of acetic acid creates a competition for the formation of coordination bonds between Cu ions with monocarboxylic acid and tricarboxylic acid [44,47]. This mechanism is shown in Figure 6, it reduces the formation of HKUST-1 nuclei for avoiding the intergrown structure so that the octahedron HKUST-1 can be well generated. Furthermore, the morphology of HKUST-1 synthesized under the optimum condition is octahedrons as presented in Figure 5g. In the EDX results shown in Figure 5h–g, the elemental composition of this optimum HKUST-1 consists of 52.28% wt. of C, 32.14% wt. of O, and 14.58% wt. of Cu, which are well distributed according to the elemental mapping with green, blue, and red color, respectively.

**Figure 7:**
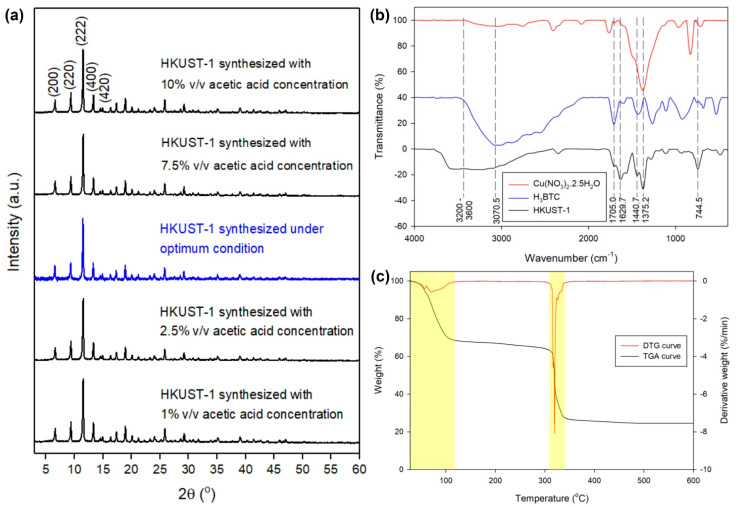
(**a**) XRD spectra of HKUST-1 synthesized under optimum condition and with various acetic acid concentrations, (**b**) FTIR spectra of HKUST-1 synthesized under optimum condition and its precursors, and (**c**) TGA-DTG curves of HKUST-1 synthesized under optimum condition.

The XRD analysis was carried out to investigate the crystallinity of HKUST-1 as patterned in Figure 7a. Here, the XRD spectra show a similar peak pattern as that obtained by the previous studies [8,9,51,57]. In Figure 7a, the XRD spectrum of HKUST-1 synthesized under the optimum condition (blue line) exhibits (200), (220), (222), (400), and (420) planes located at 2θ = 6.5°, 9.3°, 11.4°, 13.2°, and 14.9°, respectively, while the other spectra of HKUST-1 have peaks in a similar location. Furthermore, the crystallinity of HKUST-1 was calculated using Basolite C300 as the benchmark based on those five distinctive planes, where all analyzed HKUST-1 provided crystallinity higher than 100% as described in Table 4. This implies that the HKUST-1 synthesized here has higher crystallinity than commercial HKUST-1. However, HKUST-1 synthesized under the optimum condition possessed the highest $I_{200}/I_{220}$ ratio of 1.05, which was good for adsorption applications. The high $I_{200}/I_{220}$ ratio indicates that HKUST-1 has a high accessible copper active site [11]. This provides an explanation of how HKUST synthesized under the optimum condition had a high adsorption capacity of CV dye.

Figure 7b presents the FTIR spectra of HKUST-1 and its precursors, where it is seen that the peaks in the HKUST-1 spectrum originated from its precursor, namely Cu(NO_3_)_2_.2.5H_2_O and H_3_BTC. There are five main peaks in the fingerprint region of FTIR spectra, which represent Cu-O stretching at 744.5 cm^−1^, C-O stretching on carboxylate acids at 1375.2 cm^−1^, aromatic C=C stretching at 1440.7 cm^−1^, C=O stretching at 1629.7 cm^−1^, and -COO^–^ bending at 1705.0 cm^−1^ [3,15,44,58,59,60]. Outside that region, the O-H stretching vibration on carboxylate acids and O-H stretching vibration of adsorbed water are indicated by the peaks at 3070.5 and 3200–3600 cm^−1^, respectively [15,44,60]. In addition, the thermal stability of HKUST-1 was analyzed using TGA, where the result is depicted in Figure 7c. Initial weight loss occurred at less than 110 °C, indicating removal of moisture and guest molecules from the cavities of HKUST-1. In the TGA curve, a long plateau at 110–310 °C provides evidence of the good thermal stability of HKUST-1. Here, the decomposition of HKUST-1 occurred at 310–330 °C as shown by a steep descending curve and left CuO and Cu_2_O materials that were thermally stable at over 330 °C. This kind of TGA and DTG curves is also reported for proving the thermal stability of HKUST-1 in the previous studies [9,21,57,61]. It can be concluded that HKUST-1 synthesized under the optimum condition has the same thermal stability as previously reported.

### 2.3. Adsorption of Crystal Violet Dye

**Figure 8:**
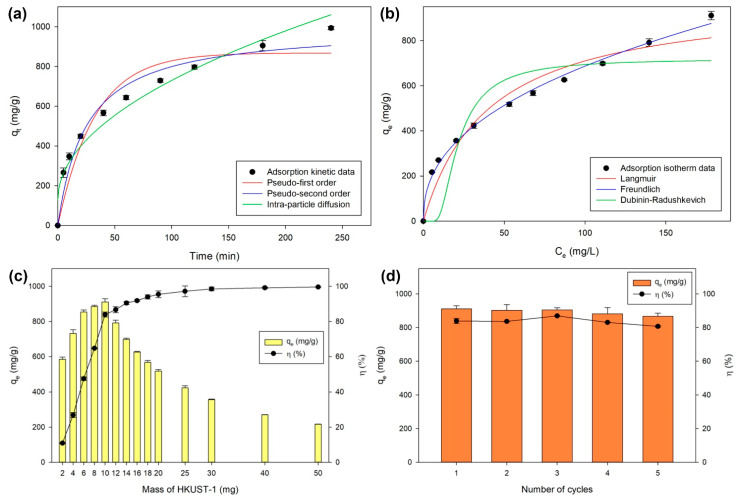
(**a**) Non-linear adsorption isotherms of CV dye onto HKUST-1, (**b**) non-linear adsorption kinetics of CV dye onto HKUST-1, (**c**) adsorption capacity and removal efficiency of HKUST-1, and (**d**) Reusability of HKUST-1.

In addition, the adsorption of CV dye onto HKUST-1 synthesized under the optimum condition was studied to understand the accessibility of this porous material. Adsorption kinetics and isotherms were investigated non-linearly as presented in Figure 8a,b. Here, the adsorption kinetic data were modelled using pseudo-first order, pseudo-second order, and intra-particle diffusion equations to reveal the adsorption behavior of CV dye on HKUST-1. Table 5 describes the constants of these modelled equations, where the $R^{2}$ of pseudo-second order equation is higher than pseudo-first order equation. This indicates that the rate-limiting adsorption mechanism of CV dye onto HKUST-1 caused by chemical interactions plays an important role compared to particle mass transfer [23,62]. Moreover, the modelled $q_{e}$ obtained from pseudo-second order equation is more in agreement with the experimental q value, where both indicate the amount of CV dye adsorbed onto HKUST-1 at the equilibrium condition. However, modelling using the intra-particle diffusion equation was also well fitted to the adsorption kinetic data as indicated by the higher $R^{2}$. The intra-particle diffusion assumes that adsorption occurs through several steps, namely external surface adsorption, liquid film diffusion, and intra-particle diffusion. Here, the C value indicates that the adsorption of CV dye onto HKUST-1 was controlled by an external diffusion mechanism due to the high C value (>>0). Adsorption controlled by the intra-particle diffusion mechanism can occur when the initial concentration of the adsorbate is low, so it will also leave an extremely low residual concentration [62]. This supports the conclusion about the mechanism controlling the adsorption of CV dye onto HKUST-1, in which a relatively high initial concentration of CV dye is used.

Furthermore, the modelling of adsorption isotherm data was conducted using Langmuir, Freundlich, and Dubinin-Radushkevich equations as presented in Figure 8b. Here, the Freundlich equation gave the best fit for the data indicated by the highest compared to the other two equations as mentioned in Table 6. This means that HKUST-1 possesses heterogeneous active sites to interact with the CV dyes. It is evidenced by the n value greater than 1, indicating an appropriate adsorption process [63]. In the Langmuir modelling, the value of $q_{max}$ as the maximum amount of CV dye adsorbed onto HKUST-1 is in agreement with the experimental q value. This CV dye adsorption is favorable because the $R_{L}$ value is between 0–1 [64]. Here, the adsorption mechanism was also figured out using the modelling of the Dubinin-Radushkevich equation. As described in Table 6, the obtained $E_{a}$ is more than 16 kJ/mol, indicating a chemisorption process [62]. Here, the chemical mechanism plays an important role because the value is far from the lower limit of 16 kJ/mol.

Simultaneously, Figure 8c shows the adsorption capacity and removal efficiency of HKUST-1 for adsorbing CV dyes. The use of 10 mg HKUST-1 on CV dye adsorption is favorable because it provides the highest adsorption capacity with the removal efficiency at the initial position towards a constant. In Figure 8d, the result of reusability is displayed to prove the feasibility of HKUST-1 in practical applications. The adsorption performance of HKUST-1 could stand up to 5 cycles with an insignificant decrease in adsorption capacity and removal efficiency. This means that HKUST-1 provides promising potential as a porous material for further applications.

## 3. Materials and Methods

### 3.1. Materials

All materials were purchased in analytical grade from Sigma-Aldrich (Singapore) and used without further purification: copper (II) nitrate hemi(pentahydrate) (Cu(NO_3_)_2_.2.5H_2_O, 98%), benzene-1,3,5-tricarboxylic acid (H_3_BTC, 95%), ethanol (C_2_H_5_OH, 99.8%), and crystal violet (CV) dye.

### 3.2. Synthesis of HKUST-1

HKUST-1 was synthesized using the room-temperature coordination modulation method, but here the addition of a certain amount of acetic acid as a modulator was given into the solution to maintain the uniform morphology of HKUST-1. Initially, Cu(NO_3_)_2_.2.5H_2_O was dissolved in 40 mL of aqueous acetic acid solution and named as solution A (0.05 M), while H_3_BTC was dissolved in 40 mL of a 1:1 ethanol-water mixture and named as solution B. After both solutions were completely dissolved, solution B was added dropwise into solution A and then the mixture was stirred using a magnetic stirrer at room temperature for a certain reaction time. Finally, the turquoise precipitate was separated from the solution by a centrifuge. It was washed twice with ethanol and dried overnight at 70 °C using an oven. In this synthesis of HKUST-1, the molar ratio of ligand to metal, reaction time, and acetic acid concentration were defined as the independent parameters (Table 7).

### 3.3. Statistical Analysis and Optimization

All statistical studies were done using Minitab 19 statistical software. The synthesis of HKUST-1 using three independent parameters was carried out with the central composite design (CCD) as the experimental design. As shown in Table 7, three desired independent parameters consisted of the molar ratio of ligand to metal, reaction time, and acetic acid concentration which were denoted as A, B, and C. Each parameter had three main middle levels and two outer extreme levels. In the synthesis of HKUST-1, all experiments were carried out following the combinations of parameters that had been designed. All experiments were replicated three times so that there were 60 experiments in total. Herein, the statistical response used was the amount of CV dye adsorbed onto HKUST-1 (q).

Furthermore, response surface methodology (RSM) was used to explore the interactions between parameters and the effects of the parameters in the synthesis of HKUST-1. Moreover, RSM provided the linear, quadratic, and two-way interaction effects of the parameters on HKUST-1 which was assessed based on the statistical response. These effects were mathematically expressed by the following Equation (2) [53,65,66]:

(2) $$q_{predicted}=\alpha_{0}+\overset{C}{\underset{i=A}{\sum }}\alpha_{i}i+\overset{C}{\underset{i=A}{\sum }}\alpha_{ii}i^{2}+\overset{C}{\underset{i=A}{\sum }}\overset{C}{\underset{j=A}{\sum }}\alpha_{ij}ij$$

where $q_{predicted}$ (mg/g) is the predicted value of q based on the statistical analysis, i and j represent the coded independent parameters (−1.68, −1, 0, +1, and +1.68) as presented in Table 7, $\alpha_{0}$ is the modelling constant, and $\alpha_{i}$, $\alpha_{ii}$, and $\alpha_{ij}$ are the coefficients of linear, quadratic, and two-way interaction, respectively. In the RSM, analysis of variance (ANOVA) was included in the investigation, where this test was used to determine the significant differences among the independent parameters.

The statistical optimization was conducted using the RSM optimization tool in the same software. From this optimization, the optimum synthesis condition was obtained based on the highest possible value of the statistical response. Next, this optimum synthesis condition was used to re-synthesize HKUST-1 for three replications. This needed to be done to validate the result of the statistical optimization.

### 3.4. Adsorption Experiments

Batch adsorption experiments of CV dye in aqueous solution were conducted at room temperature with three replications each using HKUST-1 as the adsorbent. As the statistical response, q was obtained by adsorption of 10 mL aqueous CV dye solution (1089.01 mg/L) using 10 mg HKUST-1 for a constant exposure time (24 h). These adsorption experiments were done for all synthesized HKUST-1. After that, the solids were separated from the residual solution using a centrifuge and the supernatant concentration was measured using a UV/Vis Spectrophotometer (Shimadzu Scientific Instruments Inc., Seattle, WA, USA).

Furthermore, adsorption studies were also carried out by varying the adsorption time and mass of HKUST-1 with the same other adsorption conditions. The concentrations of initial and residual aqueous CV dye solutions were also measured using a UV/Vis Spectrophotometer (Shimadzu). The amount of CV dye adsorbed onto HKUST-1 (q) and removal efficiency (η) were calculated using the following Equations (3) and (4) [67]:

(3) $$q=\frac{\left(C_{i}-C_{e}\right)V}{m}$$

(4) $$\eta =\frac{C_{i}-C_{e}}{C_{i}}\times 100\%$$

where $C_{i}$ and $C_{e}$ are the initial and residual concentrations of aqueous CV dye solution, V is the volume of aqueous CV dye solution, and m is the mass of HKUST-1 used for the adsorption. For the reusability study, HKUST-1, which has adsorbed CV dye in the first cycle of adsorption, was dried overnight at 70 °C using an oven. Next, it was washed with ethanol and water twice and then dried again before being used for the second cycle of adsorption. The subsequent adsorption was carried out the same as the previous one. These steps were repeated up to 5 adsorption cycles.

### 3.5. Characterizations

The morphology of materials was captured by a scanning electron microscopy (SEM) analysis with a JSM-6390 field emission SEM (Jeol Ltd., Tokyo, Japan) at 10 kV of accelerating voltage and 7.5 mm of working distance. The elemental composition of the materials was determined by energy-dispersive x-ray spectroscopy (EDX) analysis, which was incorporated with SEM analysis. The crystal pattern of materials was investigated by X-ray diffraction (XRD) analysis which was conducted with a PANalytical X’Pert Pro X-ray diffractometer (Philips-FEI, Eindhoven, Netherlands) using Cu Kα_1_ radiation (λ = 1.5406 Å) at 40 kV of voltage, 30 mA of current, and 0.02 °/C of step size. The crystallinity was approached by the relative intensity of five main planes towards (222) plane as the highest peak and was be calculated using the following Equation (5) [11]:

(5) $$Crystallinity\left(\%\right)=\frac{\sum_{i=1}^{5}\frac{I_{i}}{I_{222}}\;of\;sample}{\sum_{i=1}^{5}\frac{I_{i}}{I_{222}}\;of\;reference}\times 100\%$$

where $I_{i}$ is the intensity of five main planes, i.e., (200), (220), (222), (400), and (420) planes. Here, Basolite C300 as a commercial HKUST-1 was used as the reference of 100% crystallinity. The functional groups of materials were depicted by Fourier transform infrared spectrophotometer (FTIR) analysis with an FTIR Shimadzu 8400S (Shimadzu Scientific Instruments Inc., Seattle, WA, USA) under KBr pelleting method. The thermal stability and decomposition of materials were figured out by thermalgravimetric analysis (TGA) with a Perkin Elmer Diamond TG/DTA thermal analyzer (PerkinElmer Inc., Waltham, MA, USA) using 150 mL/min of nitrogen gas flow at 27.5–600 °C of the temperature range.

## 4. Conclusions

In summary, the optimum synthesis of HKUST-1 was proposed with a molar ratio of ligand to metal of 0.4703 for 27.2 h using 5% *v*/*v* acetic acid concentration. This synthesis condition was obtained from the statistical study using CCD of RSM, which showed satisfactory results. HKUST-1 synthesized under this optimum condition had the capability of adsorption of a CV dye reaching 1005.22 mg/g with a removal efficiency of 92.31%. In addition, it had a consistent octahedral morphology due to the addition of acetic acid, while its crystallinity was quite high compared to the commercial one. In CV dye adsorption, HKUST-1 proved to have a favorable adsorption mechanism with good reusability up to 5 cycles. Therefore, this optimized HKUST-1 is a promising porous material that may be used for various further applications. The present study contributes to the development of various dye adsorption or degradation systems.

## Figures and Tables

**Table 1:** Actual and predicted responses based on experiments designed with CCD of RSM.

Run Order	Blocks	Points	Parameters			Responses (mg/g)	
			*A*	*B* (h)	*C* (% *v*/*v*)	q	$q_{predicted}$
1	1	Cube	0.25	18.0	2.5	338.94	334.95
2			0.75	18.0	2.5	251.15	258.95
3			0.25	30.0	2.5	484.57	477.17
4			0.75	30.0	2.5	389.77	390.49
5			0.25	18.0	7.5	489.23	483.71
6			0.75	18.0	7.5	389.08	390.19
7			0.25	30.0	7.5	684.29	687.01
8			0.75	30.0	7.5	580.39	582.81
9		Axial	0.08	24.0	5.0	500.98	500.88
10			0.92	24.0	5.0	339.33	349.35
11			0.75	13.9	5.0	592.45	600.81
12			0.75	34.1	5.0	875.66	882.37
13			0.75	24.0	0.8	131.36	131.32
14			0.75	24.0	9.2	404.93	418.14
15		Center	0.75	24.0	5.0	976.09	972.02
16			0.75	24.0	5.0	961.66	972.02
17			0.75	24.0	5.0	965.93	972.02
18			0.75	24.0	5.0	971.97	972.02
19			0.75	24.0	5.0	970.63	972.02
20			0.75	24.0	5.0	958.03	972.02
21	2	Cube	0.25	18.0	2.5	342.49	334.95
22			0.75	18.0	2.5	265.27	258.95
23			0.25	30.0	2.5	484.69	477.17
24			0.75	30.0	2.5	396.58	390.49
25			0.25	18.0	7.5	473.75	483.71
26			0.75	18.0	7.5	396.92	390.19
27			0.25	30.0	7.5	696.10	687.01
28			0.75	30.0	7.5	569.87	582.81
29		Axial	0.08	24.0	5.0	509.95	500.88
30			0.92	24.0	5.0	354.74	349.35
31			0.75	13.9	5.0	590.16	600.81
32			0.75	34.1	5.0	901.98	882.37
33			0.75	24.0	0.8	124.22	131.32
34			0.75	24.0	9.2	427.06	418.14
35		Center	0.75	24.0	5.0	975.51	972.02
36			0.75	24.0	5.0	991.12	972.02
37			0.75	24.0	5.0	981.30	972.02
38			0.75	24.0	5.0	976.08	972.02
39			0.75	24.0	5.0	974.89	972.02
40			0.75	24.0	5.0	974.91	972.02
41	3	Cube	0.25	18.0	2.5	332.64	334.95
42			0.75	18.0	2.5	270.52	258.95
43			0.25	30.0	2.5	458.79	477.17
44			0.75	30.0	2.5	382.67	390.49
45			0.25	18.0	7.5	491.90	483.71
46			0.75	18.0	7.5	389.29	390.19
47			0.25	30.0	7.5	671.83	687.01
48			0.75	30.0	7.5	590.31	582.81
49		Axial	0.08	24.0	5.0	491.95	500.88
50			0.92	24.0	5.0	351.95	349.35
51			0.75	13.9	5.0	604.00	600.81
52			0.75	34.1	5.0	883.50	882.37
53			0.75	24.0	0.8	131.01	131.32
54			0.75	24.0	9.2	427.96	418.14
55		Center	0.75	24.0	5.0	954.05	972.02
56			0.75	24.0	5.0	972.29	972.02
57			0.75	24.0	5.0	973.07	972.02
58			0.75	24.0	5.0	978.76	972.02
59			0.75	24.0	5.0	963.96	972.02
60			0.75	24.0	5.0	976.38	972.02

**Table 2:** ANOVA for the synthesis of HKUST-1 using three independent parameters designed with CCD of RSM.

Sources	DF	Sum of Squares	Mean Squares	*F*-Value	*p*-Value	Remarks
Model	11	4,624,604	420,419	5492.81	<0.0001	Significant
Blocks	2	612	306	4.00	0.0247	Significant
A	1	83,149	83,149	1086.35	<0.0001	Significant
B	1	287,069	287,069	3750.58	<0.0001	Significant
C	1	297,919	297,919	3892.34	<0.0001	Significant
$A^{2}$	1	1,616,395	1,616,395	21,118.34	<0.0001	Significant
$B^{2}$	1	286,940	286,940	3748.90	<0.0001	Significant
$C^{2}$	1	2,627,639	2,627,639	34,330.33	<0.0001	Significant
AB	1	170	170	2.23	0.1421	Insignificant
AC	1	460	460	6.01	0.0179	Significant
BC	1	5599	5599	73.15	<0.0001	Significant
Error	48	3674	77			
Lack-of-Fit	33	2815	85	1.49	0.2072	Insignificant
Pure Error	15	859	57			
Total	59	4,628,278				

**Table 3:** Validation of optimization result.

Runs	Parameters			$q_{predicted}$ (mg/g)	q (mg/g)	Error (%)
	*A*	*B* (h)	*C* (% *v*/*v*)			
1	0.4703	27.2	5.5	1005.22	980.75	2.43
2					970.56	3.45
3					982.66	2.24
Mean q (mg/g)					977.99 ± 6.51	
Mean Error (%)					2.71 ± 0.65	

**Table 4:** Crystal information of HKUST-1 synthesized under optimum condition and with various acetic acid concentrations.

Materials	Relative Intensity					Crystallinity (%)	$\frac{I_{200}}{I_{220}}$ Ratio	Ref.
	$\frac{I_{200}}{I_{222}}$	$\frac{I_{220}}{I_{222}}$	$\frac{I_{222}}{I_{222}}$	$\frac{I_{400}}{I_{222}}$	$\frac{I_{420}}{I_{222}}$			
HKUST-1 synthesized with 1% *v*/*v* acetic acid	0.242	0.345	1.000	0.332	0.117	104.5	0.70	This study
HKUST-1 synthesized with 2.5% *v*/*v* acetic acid	0.222	0.327	1.000	0.305	0.098	100.2	0.68	
HKUST-1 synthesized under optimum condition	0.362	0.344	1.000	0.327	0.152	112.2	1.05	
HKUST-1 synthesized with 7.5% *v*/*v* acetic acid	0.253	0.326	1.000	0.307	0.110	102.5	0.78	
HKUST-1 synthesized with 10% *v*/*v* acetic acid	0.224	0.338	1.000	0.303	0.107	101.1	0.66	
Basolite C300	0.160	0.476	1.000	0.274	0.038	100.0	0.34	Mu et al. [11]

**Table 5:** Adsorption kinetic modelling constants of CV dye adsorption onto HKUST-1.

Models	Equations	Constants	Values
Pseudo-first order	$q_{t}=q_{e}\left(1-e^{-k_{1}t}\right)$	$q_{e}$ (mg/g)	868.3959
		$k_{1}$ (1/min)	0.0308
		$R^{2}$	0.9141
Pseudo-second order	$q_{t}=\frac{q_{e}^{2}k_{2}t}{1+q_{e}k_{2}t}$	$q_{e}$ (mg/g)	998.4670
		$k_{2}$ (g/mg·min)	4.0202 × 10^−5^
		$R^{2}$	0.9624
Intra-particle diffusion	$q_{t}=k_{d}\sqrt{t}+C$	$k_{d}$ (mg/g·min^0.5^)	60.1069
		C (mg/g)	128.9734
		$R^{2}$	0.9625

Note: $q_{e}$ = equilibrium adsorption capacity; $k_{1}$ = pseudo-first order rate constant; $k_{2}$ = pseudo-second order rate constant; $k_{d}$ = intra-particle diffusion rate constant; and C = constant.

**Table 6:** Adsorption isotherm modelling constants of CV dye adsorption onto HKUST-1.

Models	Equations	Constants	Values
Langmuir	$q_{e}=\frac{q_{max}K_{L}C_{e}}{1+K_{L}C_{e}}\;R_{L}=\frac{1}{1+K_{L}C_{i}}$	$q_{max}$ (mg/g)	997.3250
		$K_{L}$ (L/mg)	0.0247
		$R_{L}$	0.0358
		$R^{2}$	0.9482
Freundlich	$q_{e}=K_{F}C_{e}^{1/n\;}$	$K_{F}$((mg/g)(mg/L)^−n^)	98.6877
		n	2.3733
		$R^{2}$	0.9958
Dubinin-Radushkevich	$q_{e}=q_{max}e^{-K_{DR}\varepsilon^{2}}\;\varepsilon =RT\ln\left(1+\frac{1}{C_{e}}\right)\;E_{a}=\frac{1}{\sqrt{2K_{DR}}}$	$q_{max}$ (mg/g)	719.4296
		$K_{DR}$ (mol^2^/kJ^2^)	5.8866 × 10^−5^
		$E_{a}$ (kJ/mol)	92.1622
		$R^{2}$	0.7328

Note: $q_{max}$ = Langmuir maximum capacity; $K_{L}$ = Langmuir constant; $R_{L}$ = Langmuir separation constant; $K_{F}$ = Freundlich affinity coefficient; n = Freundlich heterogeneity constant; $K_{DR}$ = Dubinin-Radushkevich isotherm constant; and $E_{a}$ = Dubinin-Radushkevich activation energy.

**Table 7:** Levels of three independent parameters.

Parameters	Symbols	Levels				
		−1.68	−1	0	+1	+1.68
Molar ratio of ligand to metal	A	0.08	0.25	0.50	0.75	0.92
Reaction time (h)	B	13.9	18.0	24.0	30.0	34.1
Acetic acid concentration (% *v*/*v*)	C	0.8	2.5	5.0	7.5	9.2

## Data Availability

Not applicable.

## Sample Availability

Samples of the compounds are available from the authors.
